# FATP2 at the crossroads of fatty acid transport, lipotoxicity, and complex disease

**DOI:** 10.1172/JCI199873

**Published:** 2025-12-01

**Authors:** Paul N. Black, Concetta C. DiRusso

**Affiliations:** Department of Biochemistry, University of Nebraska–Lincoln, Lincoln, Nebraska, USA.

## Abstract

Type 2 diabetes mellitus affects over 38 million Americans, with diabetic kidney disease as a major complication partly driven by lipotoxicity. Fatty acid transport protein 2 (FATP2) regulates uptake and activation of long-chain fatty acids, making it a therapeutic target in metabolic disease. In this issue of the *JCI*, Khan et al. investigated FATP2 in glycemic control. In db/db mice, global FATP2 deletion reduced plasma glucose via sustained insulin secretion, with expression restricted to pancreatic α cells. FATP2-deficient db/db mice also showed suppressed glucagon and reduced alanine-stimulated gluconeogenesis, implicating α cell FATP2 in systemic glucose regulation. The FATP2-specific inhibitor lipofermata enhanced α cell–derived glucagon-like peptide 1 (GLP-1) secretion, expanded GLP-1–positive α cell mass, and promoted paracrine insulin release — effects reversed by GLP-1 receptor antagonism. These findings identify FATP2 as a key regulator linking lipid handling to α cell hormone secretion and glucose control, positioning its inhibition as a potential complement to incretin-based therapies.

## Introduction

Type 2 diabetes mellitus (T2DM) ranks as the eighth most common cause of morbidity and mortality in the United States, with diabetic kidney disease (DKD) being one of its most serious complications. A central pathological feature of DKD and other metabolic disorders is lipotoxicity, in which dysregulated fatty acid uptake and metabolism lead to aberrant lipid accumulation that causes metabolic dysregulation and death in cells of affected tissues. Among the proteins implicated in this process, fatty acid transport protein 2 (FATP2, also known as SLC27A2 or ACSVL1) has emerged as a gatekeeper that coordinates the import and metabolic activation of long- and very-long-chain fatty acids (LCFAs). Its unique biology, combined with insights from genetic deletion studies and small-molecule inhibitor development, has positioned FATP2 as both a mechanistic node and a therapeutic target in diabetes, liver disease, and cancer (1, 2).

## FATP2 and glycemic control in diabetes

**Figure 1:**
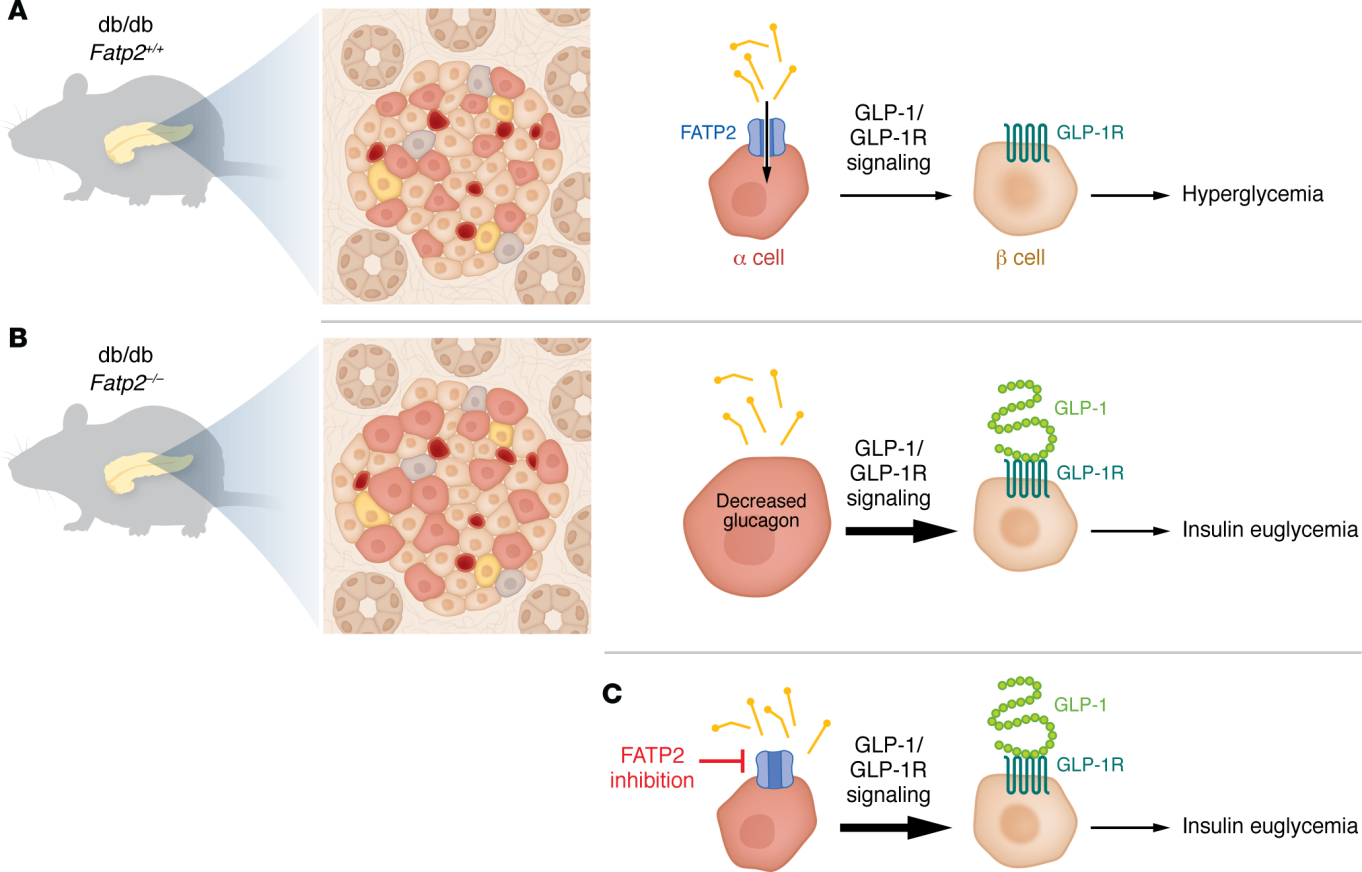
FATP2 links lipid handling to GLP-1 secretion in a cells, with implications for glycemic control. (**A**) FATP2 mediates uptake of LCFAs and very LCFAs into α cells. Khan et al. studied its effects in diabetic db/db mice, which model the β cell loss and hyperglycemia associated with T2D (4). (**B**) Global deletion of *Fatp2* in db/db mice led to α cell hypertrophy and increased GLP-1/GLP-1R signaling, which was associated with euglycemia. (**C**) Similarly, the FATP2-specific inhibitor lipofermata enhanced GLP-1 secretion in in vitro models, encouraging further investigation of FATP2 targeting as a complementary approach in diseases treated with incretin-based therapies.

Initial clues linking FATP2 to glucose homeostasis came from recent studies in T2DM-prone db/db mice reporting that global deletion of FATP2 markedly reduces plasma glucose through sustained insulin secretion (3). In work published in this issue of the *JCI*, Khan et al. performed islet analyses to demonstrate that FATP2 expression was restricted to α cells and was functional in this compartment (4). In FATP2-KO db/db mice, they observed that basal glucagon levels and alanine-stimulated gluconeogenesis were suppressed, implicating α cell FATP2 in systemic glucose regulation. Further evidence pointed to an α cell–driven glucagon-like peptide 1 (GLP-1) axis: KO mice displayed increased GLP-1–positive α cell mass, while pharmacologic inhibition of FATP2 using the FATP2-specific inhibitor lipofermata enhanced GLP-1 secretion in both αTC1-6 mouse pancreatic islet cells and human islets. Importantly, this effect on insulin release was abrogated by the GLP-1 receptor (GLP-1R) antagonist exendin(9-39)amide. By contrast, contributions from enteroendocrine GLP-1 secretion were ruled out on the basis of observations of similar glucose tolerance following oral versus intraperitoneal glucose loading, nonoverlapping expression of FATP2 and preproglucagon transcripts, and absence of FATP2 and GLP-1 colocalization in intestinal tissues. Together, these findings reveal that FATP2 deletion or inhibition lowers glucose by stimulating α cell–derived GLP-1 secretion and paracrine insulin release (Figure 1), offering a mechanistic complement to incretin-based therapies (4).

## Molecular structure and isoforms of FATP2

At the molecular level, FATP2 functions as both a transporter and an enzyme. It facilitates the uptake of LCFAs and very LCFAs at the plasma membrane and has very-long-chain acyl-CoA synthetase (VLCS) activity (5, 6). Two human splice isoforms provide functional separation of these roles. FATP2a, the full-length form, retains acyl-CoA synthetase activity and supports fatty acid transport, whereas FATP2b, which lacks exon 3 that contains the adenylate-forming region of the enzyme, is catalytically impaired but remains capable of transporting fatty acids (7). Subcellular localization studies suggest that FATP2 localization is likely to be isoform biased: FATP2a is enriched in both the ER and peroxisomes, where it activates very-long-chain substrates for β-oxidation, while FATP2b appears to be largely restricted to the ER and plasma membrane (7, 8). The identification of a naturally occurring splice variant, FATP2b, that functions only in transport underscores the concept of vectorial acylation, in which FATP2 and a cognate acyl-CoA synthetase functionally cooperate to maintain low cytosolic fatty acid concentrations and drive net influx of exogenous fatty acids (8, 9).

## Mechanistic insights into FATP2 function

Full-length FATP2 contains conserved adenylate-forming and FATP/VLACS motifs that are cytoplasmically oriented to couple uptake to downstream metabolism (6). In hepatocytes, most FATP2 resides outside peroxisomes and facilitates LCFA import, but the smaller peroxisomal pool accounts for peroxisomal VLCS activity (5). FATP2 also interacts with ceramide synthase 2 (CerS2), linking fatty acid uptake and activation to sphingolipid and ether lipid synthesis pathways (10).

Importantly, work in FATP2-KO mice has revealed that deletion of FATP2 in the liver activates a compensatory program mediated by PPARα, remodeling the hepatic transcriptome toward enhanced fatty acid oxidation and catabolism (11). These changes included increased expression of genes involved in β-oxidation, peroxisomal metabolism, and detoxification pathways, while decreasing lipid storage and stress-related transcripts. This study highlights the dual role of FATP2 in determining whether fatty acids are directed toward catabolic versus lipotoxic pathways. By serving as a molecular switch between uptake, activation, and downstream routing, FATP2 directly links fatty acid entry to broader transcriptional and metabolic networks that govern cellular lipid homeostasis (11).

## FATP2 in DKD

FATP2 is the predominant fatty acid transporter in renal proximal tubular cells, positioned at the apical membrane to capture albumin-bound, nonesterified fatty acids from the glomerular filtrate (9). In DKD, excessive fatty acid uptake through FATP2 induces tubular lipotoxicity, apoptosis, and fibrosis (12). Genetic deletion of FATP2 in diabetic mouse models improves albuminuria, reduces tubular lipid accumulation, preserves histology, and lowers fasting glucose levels, highlighting a vicious cycle between proximal tubule lipotoxic stress and systemic glucolipotoxicity (3). Pharmacologic inhibition with the FATP2 inhibitor lipofermata reduces tubular lipid deposition and fibrotic marker expression while restoring fatty acid oxidation programs, thereby interrupting DKD progression (13).

## FATP2 in liver lipotoxicity and steatotic liver disease

The liver expresses FATP2, which channels exogenous fatty acids into glycerolipid and signaling pools. Deletion of FATP2 remodels the hepatic transcriptome toward PPARα-regulated fatty acid catabolism and reduces lipid burden (7, 11). Conversely, expression of FATP2 under high-fat conditions promotes excess lipid storage, whereas pharmacologic inhibition using lipofermata protects hepatocytes from palmitate-induced lipotoxicity and reduces dietary fat uptake (9).

## FATP2 in cancer

FATP2 is also emerging as a critical immunometabolic regulator in cancer. In polymorphonuclear myeloid-derived suppressor cells (PMN-MDSCs), tumor-derived GM-CSF/STAT5 signaling upregulates FATP2, enhancing the arachidonic acid uptake and prostaglandin E_2_ synthesis that suppress T cell responses. Genetic or pharmacologic FATP2 blockade abolishes MDSC-mediated immunosuppression, reduces tumor growth, and synergizes with checkpoint inhibitors (14). Additionally, tumor-intrinsic FATP2 overexpression, as observed in differentiated thyroid carcinoma, promotes proliferation and invasion via MAPK signaling, and silencing FATP2 reduces tumor aggressiveness (15).

## Discovery of lipofermata via high-throughput screening

The development of FATP2 inhibitors identified using a high-throughput chemical screen designed to isolate compounds that block LCFA transport without interfering with acyl-CoA synthetase activity provided a valuable chemical tool to study FATP2 activity (16, 17). Using a humanized yeast strain lacking genes for fatty acid transport (*FAT1*) and activation (*FAA1*) while expressing human FATP2b, more than 100,000 small molecules were evaluated for their ability to inhibit uptake of the fluorescent LCFA analog C_1_-BODIPY-C_12_ (14). This primary screen identified 234 hits, of which five chemotypes were validated in human Caco-2 and HepG2 cells that endogenously express FATP2. These compounds inhibited LCFA uptake with low-micromolar potency while having only modest activity in 3T3-L1 adipocytes, which predominantly express FATP1 (17). Importantly, these inhibitors did not impair cell viability, barrier integrity, glucose transport, or long-chain acyl-CoA synthetase activity, confirming their specificity for fatty acid transport. Among these, 5′-bromo-5-phenyl-spiro[3H-1,3,4-thiadiazole-2,3′-indoline]-2′-one, later named lipofermata, emerged as the lead selective FATP2 transport inhibitor (9, 16, 17). Subsequent work demonstrated that lipofermata acts as a noncompetitive inhibitor of FATP2, effectively blocking LCFA uptake while having no effect on medium-chain fatty acid diffusion or long-chain acyl-CoA synthetase activity (9, 17).

## Therapeutic implications and unifying model

Taken together, the evidence presented by Khan et al. and other groups positions FATP2 as a molecular gatekeeper at the interface of fatty acid transport, activation, and disease. At the plasma membrane and ER, FATP2a couples LCFA uptake with activation, whereas FATP2b supports transport without intrinsic enzymatic activity (6–9). In peroxisomes, FATP2a activates very-long-chain substrates for β oxidation, with deletion of the protein that reduces VLCS activity but triggering compensatory PPARα pathways (5, 11). The therapeutic implications are broad: pharmacologic blockade of FATP2 with compounds such as lipofermata can protect parenchymal tissues like the kidney and liver from lipid overload, while also reprogramming the tumor microenvironment by disarming immunosuppressive MDSCs (9, 13, 14). Moreover, the link between the FATP2 inhibition, α cell GLP-1 secretion, and improved glycemic control described by Khan and colleagues extends FATP2 biology into the endocrine pancreas and provides a complementary mechanism to incretin-based therapies (4). By selectively targeting fatty acid transport while sparing activation, FATP2 inhibitors exemplify a rational pharmacologic approach to disentangle lipid uptake from metabolism in diverse pathological contexts.

## Funding support

This work is the result of NIH funding, in whole or in part, and is subject to the NIH Public Access Policy. Through acceptance of this federal funding, the NIH has been given a right to make the work publicly available in PubMed Central.

- NIH grants RO1-DK07076 and RO1-GM56850 (to PNB and CCD).
- University of Nebraska (Research Initiative Proof of Concept and Agricultural Research Division, to PNB and CCD).

## Version 1 12/01/2025

Electronic publication
